# Donors’ Outcome After Living Donor Liver Transplantation in a University Teaching Hospital: A Case Series

**DOI:** 10.7759/cureus.71858

**Published:** 2024-10-19

**Authors:** Narendra Maharjan, Deepak Sharma, Sumita Pradhan, Bishnu P Kandel, Paleswan Joshi Lakhey, Ramesh S Bhandari

**Affiliations:** 1 Department of Surgical Gastroenterology, Maharajgunj Medical Campus, Institute of Medicine, Tribhuvan University Teaching Hospital, Kathmandu, NPL

**Keywords:** donor hepatectomy, end-stage liver disease, future liver remnant, living donor liver transplantation, quality of life

## Abstract

Background

Liver transplantation is the standard treatment for end-stage liver disease. Living donor liver transplantation is more commonly performed in Asian countries as compared to the Western due to the lack of organ donation. Donor safety is the key to sustaining a liver transplant program. Thus, we aimed to evaluate the overall safety of living donors and health-related quality of life using the 12-item Short Form Health Survey (SF-12) questionnaire at our institution.

Methodology

We analyzed the medical records of patients who underwent donor hepatectomy at Tribhuvan University Teaching Hospital, Kathmandu, from May 31, 2019, to April 18, 2023. Demography, postoperative complications, and quality of life were analyzed.

Results

The mean age of the 10 live liver donors was 27.9 years. Half of them were male. One of them had a post-hepatectomy bile leak and others did not have any post-operative complications. They have good physical and mental health status after liver donation as indicated by the average physical component summary and mental component summary scores of more than 50.

Conclusion

The case series highlights the safety and favorable outcomes of liver donors at a low-volume liver transplant center, where stringent preoperative assessments and careful surgical techniques were employed.

## Introduction

End-stage liver disease is one of the major health problems in Nepal and alcohol is the most common etiology [1,2]. Liver transplantation is an established and the only treatment option for this disease. In comparison to the Western world where deceased donor liver transplantation (DDLT) is more commonly performed, living donor liver transplantation (LDLT) accounts for 90% of liver transplantation in most Asian countries [3]. The donor's safety and outcome are the key to a successful LDLT program in any institution. Since the first successful living donor liver transplant [4,5], many centers have performed the procedure with excellent outcomes. However, the safety of liver donors remained a major concern as severe complications including mortality have been reported [6]. Also, there is a general belief that performing LDLT with acceptable outcomes in a low-volume center is hard. Living donor liver transplantation was started at our institution on May 31, 2019, and performed 10 liver transplantations till April 18, 2023. Therefore, we will evaluate the overall safety of living donors and health-related quality of life using the 12-item Short Form Health Survey (SF-12) questionnaire at our institution.

## Materials and methods

A retrospective analysis of medical records of all the live liver donors, who underwent surgery at the Department of Surgical Gastroenterology, Tribhuvan University Teaching Hospital, Nepal, from May 31, 2019, to April 18, 2023, was conducted, and follow-up data were collected till May 31, 2024. The postoperative complications were graded according to the Clavien-Dindo classification. Statistical analysis was performed using SPSS version 20 (IBM Corp., Armonk, NY, USA).

All voluntary donors underwent investigations and assessments in a staged manner to confirm fitness for liver donation. This included independent assessment by multiple departments including hepatology, cardiology, anesthesiology, psychiatry, dentistry, nephrology, ophthalmology, otolaryngology, and endocrinology. A meeting was then held with the patient and his/her relatives to explain the whole process, the risks involved in donor hepatectomy, and regarding returning to normal activity and work. Surgery was scheduled after obtaining approval from the liver transplant approval committee of our institution.

Preoperative donor evaluation

The inclusion criteria for donors were age between 18 and 55, abstinence from alcohol for at least six months and absence of diabetes. Triphasic Contrast Enhanced Computed Tomography (CECT) abdomen was done for volumetric assessment and planning for surgery while plain Computed Tomography (CT) abdomen was used to calculate the Liver Attenuation Index (LAI). LAI less than 5 for right or left liver graft and less than 0 for lateral section graft were rejected for donation. Portal vein anatomy and hepatic artery anatomy were classified according to Nakamura’s and Michel’s classifications respectively [7,8]. A liver biopsy was not routinely done. Magnetic resonance cholangiopancreatography (MRCP) was done to assess bile duct anatomy and classify it according to Huang's classification [9]. The nutrition risk index (NRI) [10] was used to assess the donors' nutritional status. It was calculated using the online calculator https://www.mdcalc.com.

Surgical procedure

All donors received injections of ceftriaxone prophylactically. Modified Makuuchi skin incision was routinely used. Inflow and outflow structures were isolated and looped. Liver parenchymal transection was done using Cavitron Ultrasonic Suction and Aspirator (CUSA). An intraoperative cholangiogram was performed before and after bile duct transection to determine the line of transection and bile leakage respectively.

Postoperative management

Donors were transferred to the liver transplant Intensive Care Unit after completion of hepatectomy. Doppler ultrasound was performed routinely on the first postoperative day. They were usually moved to the ward on day two. The length of hospital stay was calculated from the day of surgery to the day of discharge. An ultrasound of the abdomen, liver function test, and chest X-ray were performed on a day before discharging the donor. They were followed up weekly for the first month after surgery. Then they were followed up at three, six and 12 months and annually.

The health-related quality of life of the donors was assessed using the SF-12 version 1.0 [11]. There are two summary scores calculated in SF-12. They are mental component scores and physical component scores. The scores were calculated using an online calculator: https://orthotoolkit.com/sf-12/. The questionnaire of the SF-12 was mailed to all the donors and responses were collected. 

## Results

There were 10 live donors in the study. The mean age of them was 27.9 years. Half of them were male. None were overweight or obese and their nutritional status was good. All donors were immediate family; daughter and son were the most common donors. Their liver quality based on LAI was good. None of them underwent liver biopsy. All the donors underwent right hepatectomy without incorporation of middle hepatic vein except one who donated a lateral section to his 11-month-old son (Table 1). One of them had a post-hepatectomy bile leak and others do not have any post-operative complications. The bile leak was detected in an intraoperatively placed drain and subsided spontaneously. They have good physical and mental health status after liver donation as indicated by the average physical component summary and mental component summary scores of more than 50 (Table 2).

**Table 1:** Demography and preoperative characteristics of donors BMI: Body Mass Index, NRI: Nutrition Risk Index, MRH: modified right hepatectomy

Parameters		Values (n=10)
Age, years (mean ± SD)		27.9± 4.5
Sex (male: female)		5:5
BMI, kg/m^2^(mean ± SD)		22.1 ± 2.6
NRI, mean ± SD		109.6± 3.6
Relation to recipient, n (%)	Daughter	3 (30)
	Son	3 (30)
	Wife	2 (20)
	Son-in-law	1 (10)
	Father	1 (10)
Graft type, n (%)	MRH	9 (90)
	Lateral section	1 (10)
Portal vein, n (%)	Nakamura A	7 (70)
	Nakamura B	2 (20)
	Nakamura D	1 (10)
Bile duct, n (%)	Huang A1	2 (20)
	Huang A2	1 (10)
	Huang A3	3 (30)
	Huang A4	3 (30)
Hepatic artery, n (%)	Michel 1	6 (60)
	Michel 3	1 (10)
	Michel 5	1 (10)
	Michel 6	1 (10)
	Michel 11	1 (10)
Future liver remnant (mean ± SD)		43 ± 14.5
Liver attenuation index (mean ± SD)		11.7 ± 4

**Table 2:** Perioperative Outcome and Quality of life of donors PCS: Physical Component Summary, MCS: Mental Component Summary, SF 12: Short Form 12 (PCS and MCS scores above 50 indicate a better-than-average health-related quality of life, and scores below 50 suggest below-average health)

Parameters			Values (n=10)
Operative time, min (mean ± SD)			502 ±119
Postoperative complication, n (%)		Bile leak	1 (10)
		None	9 (90)
Length of hospital stay, days (mean ± SD)			10±1.7
Clavien-Dindo Grade, n (%)	0		9 (90)
	1		1 (10)
SF 12	PCS, mean ± SD		50.23± 5.96
	MCS, mean ± SD		56.24 ± 4.47

## Discussion

Live liver donation is more common in Eastern countries as compared to Western. This may be due to a lack of or inadequate pool of deceased donations. Donor surgery is one of the complex surgeries and is associated with various postoperative complications [12]. The rate of postoperative complications reported in the literature varies widely, ranging from 18 to 22% [13]. In this study of 10 liver donors, there was one (10%) donor who had post-operative bile leakage detected in an intraoperatively placed abdominal drain. The bile leak decreased with time and stopped spontaneously over seven days. It has been reported that bile leakage is typically detected on early postoperative days and its incidence ranges from 5 to 15% [14]. Ruh et al. identified that the rate of bile leak in donors increases when a margin is less than 5 mm from the main duct and the presence of multiple hepatic arteries [15]. This is because less margin from the main duct increases the chance of injury to it and the presence of multiple hepatic arteries requires extensive dissection, leading to ischemic injury to the bile duct.

It is of paramount importance to decrease donor postoperative morbidity. For this, preoperative evaluation plays a vital role. Age, body mass index (BMI), future liver remnant (FLR), and LAI were shown to affect the postoperative outcome of liver donors.

There is no well-defined upper age limit for live liver donation. However, most of the centers accept 50 to 60 years as an upper age limit [13]. In this study, the oldest liver donor was 35 years old.

Hepatic steatosis is associated with poor liver graft function and recipient outcome. Although liver biopsy is the gold standard for the estimation of steatosis, it is an invasive procedure and is associated with various complications [16]. So, non-invasive methods like BMI and LAI have been used for the assessment.

BMI is considered a predictor of hepatic steatosis which has an adverse effect on outcomes of donor and recipient undergoing LDLT. It has been found that patients with a BMI less than 25 had no hepatic steatosis, while a BMI between 25 to 28 and greater than 28 had hepatic steatosis in 33.3% and 76% of patients respectively [17]. However, BMI alone cannot determine the outcome of the donor. Donors having a BMI between 30 and 35 without liver steatosis >10% and other comorbidities associated with obesity such as diabetes, dyslipidemia, and hypertension, had undergone liver transplant surgery safely [18]. However, non-obese donors were included in this study for liver donation.

The liver attenuation index is the difference between liver and spleen attenuation on non-contrast CT. An ideal liver donor should have hepatic steatosis of less than 20% which corresponds to LAI ≥ 6. LAI ≤ - 5 corresponds to steatosis of > 30%, which is a contraindication for live liver donation. Also, LAI between -5 and 5 is an indication for liver biopsy to accurately determine the degree of steatosis [19]. In this study, a liver graft having LAI ≥ 6 was used in all cases.

Future liver remnant is an important factor to be considered during donor selection. Though some reports suggest an FLR of less than 30% can undergo liver donation safely, most centers prefer it to be more than 30-35% to maximize donor safety [20]. In this present study, the mean FLR was 43%.

Thus, it seems that our institute is very stringent at donor selection to increase donor safety, as it is the beginning phase of the liver transplantation program and donor safety is the key to sustaining the liver transplantation program.

Besides postoperative outcomes, it is paramount for liver donors to have a good quality of life following donor hepatectomy. The quality of life can be measured by various tools. However, SF-12, a 12-item health survey, is a validated tool for the assessment. It helps to measure physical and mental health status in terms of Physical Component Summary (PCS) and Mental Component Summary (MCS) scale scores. These scores have undefined upper and lower limits. However, the average PCS and MCS scale scores of the general US population were estimated to be 50 with a standard deviation of 10 [21]. The average PCS and MCS scores of liver donors in our study were also above 50, suggesting that the donors have good physical and mental health status after liver donation. There are some limitations in this study. It is a single-center, retrospective study and the sample size is small. The SF-12 questionnaire is a validated method of assessing health-related quality of life, but we did not have the donors' pre-donation quality of life. So, it is hard to conclude how much the surgery has impacted their quality of life.

## Conclusions

The case series highlights the safety and favorable outcomes of liver donors at a low-volume liver transplant center, where stringent preoperative assessments and careful surgical techniques were employed. Despite a single case of post-hepatectomy bile leak, which resolved without further complications, all donors demonstrated good physical and mental health postoperatively, as indicated by SF-12 health survey score above 50. This suggests that with meticulous donor selection and surgical care, living donor liver transplantation can be conducted safely, even in lower-volume centers, contributing to the sustainability of liver transplantation programs.
